# Advances in the Prevention of Cervical Cancer by Anti‐Human Papillomavirus Agents

**DOI:** 10.1002/cam4.70847

**Published:** 2025-04-06

**Authors:** Hangdi Chen, Kai Guo, Zhihao Bai, Liuyi Lu, Bin Liu, Jiali Zhang, Meiyin Zhong, Changfen Xu, Wanghuan Chen, Aiwu Huang, Yuemin Ding

**Affiliations:** ^1^ Department of Clinical Medicine School of Medicine, Hangzhou City University Hangzhou China; ^2^ Department of Obstetrics and Gynecology Hangzhou Lin'an District Hospital of Traditional Chinese Medicine, Affiliated Hospital of Hangzhou City University Hangzhou China

**Keywords:** anti‐HPV agent, antivirus, cervical cancer, HPV, prevention and treatment

## Abstract

**Background:**

Cervical cancer remains a major global health threat for women, primarily driven by human papillomavirus (HPV) infection. While HPV vaccination serves as the cornerstone of prevention, disparities in vaccine accessibility persist across low‐income countries. Secondary prevention through screening faces challenges in public engagement, often leading to late‐stage diagnoses. Recent advancements in novel anti‐HPV drugs offer expanded opportunities for cervical cancer management.

**Aim:**

This review examines emerging anti‐HPV therapeutics to provide insights into innovative strategies for cervical cancer prevention and treatment.

**Methods:**

We conducted a systematic analysis of published studies investigating anti‐HPV agents, focusing on their molecular mechanisms and clinical efficacy in cervical cancer prevention.

**Results & Conclusions:**

Multiple promising anti‐HPV agents have been identified, including 3‐hydroxyphthalic anhydride‐modified bovine β‐lactoglobulin (3HP‐β‐LG), carrageenan, defensins, and 25‐hydroxycholesterol (25HC). These compounds exert antiviral effects through distinct mechanisms: 3HP‐β‐LG competitively inhibits viral attachment, carrageenan blocks HPV entry via heparan sulfate mimicry, defensins inhibit the dissociation of viral capsid, and 25HC activates cholesterol‐mediated antiviral pathways. They have demonstrated strong inhibitory effects on HPV infection, making them novel therapeutic candidates for the prevention and treatment of cervical cancer.

## 1 Introduction

Cervical cancer ranks as the fourth most prevalent cancer among women globally, remaining a major threat to women's health around the world [1]. In 2020, the global cancer epidemiology database GLOBOCAN established by the World Health Organization showed that 604,000 women worldwide were diagnosed with cervical cancer, and 342,000 women died from the disease [2]. Cervical cancer can primarily be categorized into adenocarcinoma and squamous cell carcinoma, with HPV infection identified as the leading risk factor [2]. Furthermore, HPV infection influences the prognosis and outcomes of cervical cancer. Patients with HPV‐positive cervical cancer generally experience more favorable treatment results and better prognoses than those with HPV‐negative cervical cancer. In the study conducted by Carunchio et al. the overall survival (OS) rate for patients with HPV‐negative cervical cancer was reported to be 67.7 months, with a 95% confidence interval (CI) of 20.0–106.9, whereas the OS for HPV‐positive patients was recorded at 108.9 months, with a 95% CI of 97.7–120.0 [3]. A separate meta‐analysis encompassing 2838 cervical cancer cases from 17 studies indicated that the presence of HPV DNA correlates with a positive prognosis for cervical cancer patients (overall survival (OS): pooled hazard ratio (HR) = 0.610, 95% CI = 0.457–0.814, *p* = 0.001; disease‐free survival (DFS): pooled HR = 0.362, 95% CI = 0.252–0.519, *p* < 0.001) [4]. Currently, the prevention of cervical cancer primarily relies on HPV vaccination and cervical cancer screening. However, access to HPV vaccines is uneven, and many low‐ and middle‐income countries have yet to implement effective primary vaccination strategies. Additionally, public participation in cervical cancer screening initiatives is subpar. Fortunately, recent advancements in various methods to combat HPV infections offer new avenues for the prevention of cervical cancer. For example, the clinical validation of bioadjuvants prepared from 3‐hydroxyphthalic anhydride‐modified bovine β‐lactoglobulin (3HP‐β‐LG) has proven safe and effective, and has been approved by the China Food and Drug Administration (CFDA) for the prevention and treatment of cervical HPV infections [5, 6]. Moreover, studies have indicated that carrageenan, defensins, and 25‐hydroxycholesterol also demonstrate anti‐HPV effects and may become potential solutions for HPV infection prevention [7, 8, 9, 10].

### 1.1 Conventional Prevention Strategies

Cervical cancer is a preventable malignancy. HPV infection is an important pathogenic factor of cervical cancer. Most cases of cervical cancer are related to high‐risk HPV infection. When high‐risk HPV infects the cervix, it can cause low‐grade precancerous lesions, such as cervical intraepithelial neoplasia grade 1 (CIN 1), which many individuals can clear through their immune responses. However, persistent infection may progress to more severe precancerous conditions, like CIN 2/3 and adenocarcinoma in situ (AIS), which have the potential to develop into invasive cervical cancer. Therefore, effective strategies for preventing and treating HPV infections are crucial for significantly lowering the global incidence of cervical cancer [11, 12]. Prevention can be achieved through primary prevention, such as prophylactic HPV vaccination and secondary prevention measures, including cervical cancer screening and the treatment of precancerous lesions [13]. Most cervical cancers can be prevented and treated if the vaccine is effectively administered and regular screening is performed. Furthermore, upon early detection, even with the presence of tumors, patients can anticipate a relatively lengthy survival period, coupled with a favorable prognosis and preserved quality of life [14]. Currently, standard cervical cancer prevention strategies include prophylactic HPV vaccination, cervical cancer screening, and lifestyle improvements, etc.

### 1.2 Prophylactic HPV Vaccination

Inducing the production of neutralizing antibodies is the basis for the action of prophylactic HPV vaccines [15]. Currently, HPV vaccines consist of virus‐like particles (VLP) assembled from recombinant HPV capsid proteins, which can induce the body to produce different types of neutralizing antibodies. These neutralizing antibodies can bind directly to HPV or reduce infection by inhibiting cellular uptake of HPV. Because these vaccines do not contain viral DNA, they are non‐infectious [15]. So far, there are primarily three types of HPV vaccines on the market: bivalent, quadrivalent, and nine‐valent, covering high‐risk types like HPV16 and HPV18, and these vaccines have shown excellent results in preventing cervical cancer [16]. Moreover, research by Bogani et al. indicates that HPV vaccination can still prevent the development of lower reproductive tract lesions, such as epithelial neoplasia of the anus, vulva and vagina, even in women who have undergone hysterectomy [17]. However, HPV vaccines are not without safety concerns, such as pain at the injection site, fever, and soreness in joints and muscles [15]. Additionally, the vaccine's limitations should not be ignored either. Although many people have been vaccinated, the overall population coverage of HPV vaccines remains low. For developing countries, the high cost of vaccines makes them unaffordable for many low‐income groups, and challenges like vaccine refrigeration and transportation also exist, resulting in extremely unbalanced vaccination rates between developing and developed countries [15].

### 1.3 Cervical Cancer Screening

The purpose of cervical cancer screening is to identify precancerous lesions or cancer in its early stages and decrease the incidence and mortality of cervical cancer through timely intervention. The United States Preventive Services Task Force (USPSTF) advises cytology screening every 3 years for women aged 21–29, and high‐risk HPV testing every 5 years for women aged 30–65 [18]. Women at high risk of precancerous cervical lesions, including those with abnormal cervical cells or who are immunocompromised, should participate in screening more frequently. Screening can stop at age 65 for those who have been adequately screened previously, especially if they have had 10 years of negative results and no high‐risk factors. Additionally, patients who have had a hysterectomy without specific high‐risk factors can also stop screening [19]. Currently, the clinical screening methods include the Pap smear test, HPV DNA testing, visual inspection with acetic acid, and colposcopy. In cases of positive screening results, such as the detection of abnormal cells in the Pap smear, a colposcopy or even a biopsy should be performed to determine if there are precancerous lesions. If precancerous lesions are identified, surgery can be used to remove the abnormal cells to prevent further development of cervical cancer. Treatment of precancerous lesions can prevent cancer in over 90% of cases [19].

### 1.4 Lifestyle Improvements

Research indicates that dietary factors can significantly affect the progression of HPV: eating more fruits, vegetables, and antioxidants can lower the risk of HPV infection, while a high intake of animal fats and high‐calorie foods can increase the risk. Smoking is also a risk factor for cervical cancer. Quitting smoking is beneficial for the prevention and treatment of cervical cancer. Obesity is another high‐risk factor for HPV infection mortality. A study in the United States found that a Body Mass Index (BMI) ≥ 35 increases the likelihood of dying from cervical cancer, underscoring the importance of regular exercise and weight management in reducing this risk. Women with obesity should be more frequently screened for cervical cancer [20, 21].

### 1.5 New Prevention Strategies

HPV infection is the foremost cause of cervical cancer. Given the limited availability of global resources aimed at combating HPV, researchers aspire to unearth novel medications that can inhibit HPV infection, thereby addressing the gaps in vaccination and cervical cancer screening initiatives. Currently, bovine β‐lactoglobulin modified with 3‐hydroxyphthalic anhydride has been used in clinical treatment [5]. Furthermore, various bioactive compounds, including carrageenan, defensins, and 25‐hydroxycholesterol, have demonstrated significant antiviral properties against HPV, indicating their potential as therapeutic or preventative agents [7, 8, 9, 10].

### 1.6 3HP‐β‐Lg

In 1996, research indicated that 3HP‐β‐LG demonstrated inhibitory effects on a variety of enveloped viruses, including the human immunodeficiency virus (HIV) [22]. Advancing this research, in 2013, Jiang et al. uncovered the potent antiviral properties of bovine β‐lactoglobulin against non‐enveloped viruses, notably HPV subtypes 16, 18, and 6. They proposed that its mechanism might involve the negatively charged residues of 3HP‐β‐LG interacting with the positively charged regions of HPV L1 protein's C‐terminal and L2 protein's N‐terminal regions to inhibit HPV infection [23]. Subsequently, a randomized, open‐label clinical trial was conducted involving 77 individuals infected with high‐risk HPV, who were randomly assigned to either a JB01‐BD treatment group (*n* = 38) or a blank control group (*n* = 39). Those in the JB01‐BD group received vaginal doses of JB01‐BD (3 g) every other day for 3 months, excluding menstruation, while the control group did not receive any treatment. Post‐treatment analysis revealed a significant increase in the proportion of positive to negative HPV cases, with only 13.5% (5/37) in the untreated group compared to 60.5% (23/38) in the treated group (*p* < 0.001). No serious adverse events were reported, confirming the treatment's safety and effectiveness [5, 24].

**FIGURE 1:**
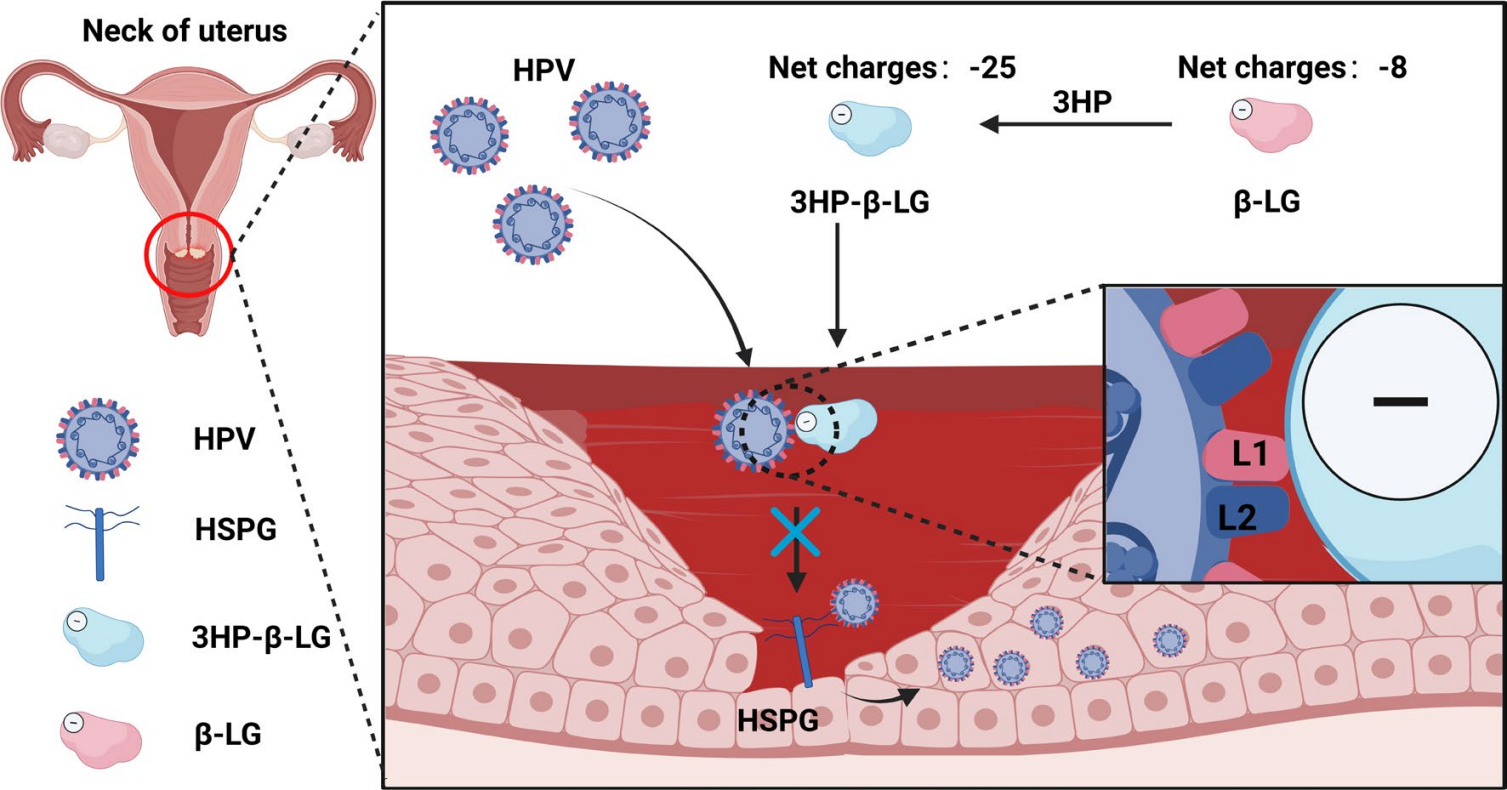
The mechanism of 3‐hydroxyphthalic anhydride‐modified bovine β‐lactoglobulin (3HP‐β‐LG) inhibiting HPV infection. HPV enters the basal layer through wounds on the vaginal mucosa, binds to heparan sulfate proteoglycan (HSPG) receptors on the basement membrane through L1 protein, enters mucosal epithelial cells, and replicates within them. After 3HP modification, the net negative charge of β‐LG changed from −8 to −25, which can generate electrostatic interactions with the positive charge region of HPV L1 protein, blocking the binding of L1 protein to HSPG receptors and inhibiting HPV from entering and infecting cells.

In 2019, Chen et al. further elucidated the specific mechanism underpinning 3HP‐β‐LG's antiviral activity. HPV tightly binds to its receptors such as heparan sulfate proteoglycan (HSPG) on the basement membrane of host cells through L1 protein, mediating virus entry and infection of host cells [25]. Li et al. discovered that neutralizing antibodies binding to the L1 protein can inhibit HPV infection, identifying L1 protein as a critical target for anti‐HPV infection [26]. Chen et al. found that β‐LG modified with HP, which changes its net charge to −25, can bind to HPV L1 protein through its negatively charged region interacting with positively charged peptides on HPV L1 protein, thereby interfering with the binding of HPV to HSPG receptors on the vaginal mucosa's basement membrane, ultimately preventing HPV from entering and replicating in mucosal epithelial cells (Figure 1). Meanwhile, Chen et al. also found that this binding may be non‐covalent and reversible [25].

### 1.7 Carrageenan

**FIGURE 2:**
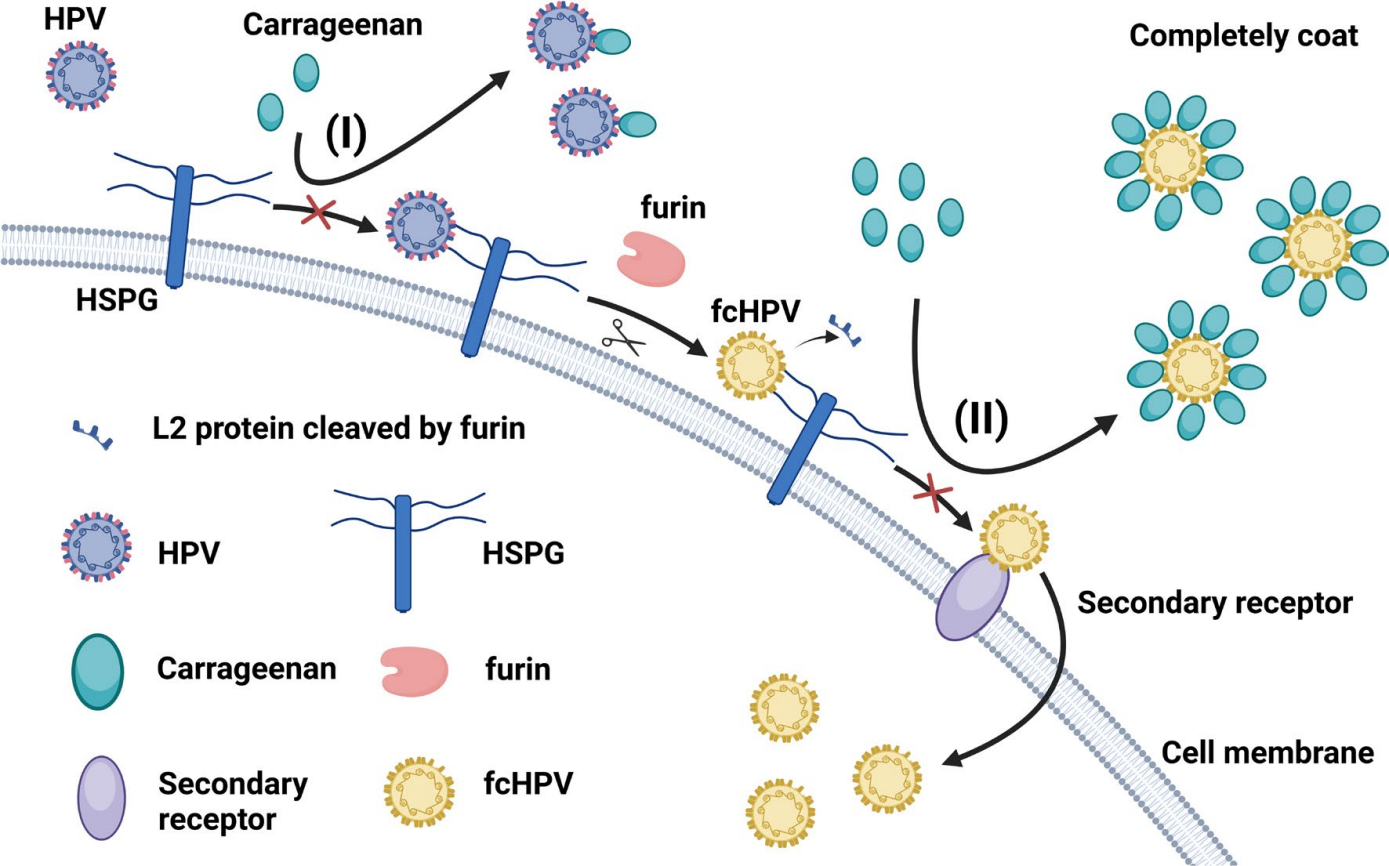
The mechanism of carrageenan inhibiting HPV infection. After HPV binds to heparan sulfate proteoglycan (HSPG), it is cleaved by furin protease and then transferred to secondary receptors, ultimately entering the cell. (I) Carrageenan can competitively bind to HPV by chemically mimicking heparan sulfate (HS), thereby blocking the initial binding of HPV to cells and preventing infection. (II) At the same time, carrageenan also has a secondary inhibitory effect. After furin‐cleaved HPV (fcHPV) binds to cells, high concentrations of carrageenan completely envelop the virus and mask the surface of the virus that binds to secondary receptors, thereby blocking HPV binding to secondary receptors and preventing infection.

Carrageenan, a water‐soluble linear sulfated polysaccharide obtained from edible red algae, is widely used in experimental medicine and the pharmaceutical industries [27, 28]. Its safety has been validated by the US Food and Drug Administration (FDA) [28]. Current research has proven that carrageenan exhibits a broad spectrum of antiviral properties, inhibiting various enveloped viruses such as herpes simplex virus, cytomegalovirus, HIV, influenza virus, and hepatitis virus [27, 29, 30, 31, 32, 33, 34]. In 2006, Christopher B. Buck et al. revealed that carrageenan is also a potent inhibitor of HPV [35]. Many sulfated polysaccharides are believed to inhibit viral infection by competitively binding to the HPV capsid with heparan sulfate. Carrageenan, as a sulfated polysaccharide, can also inhibit infection by binding to the viral capsid (Figure 2) [35]. The three main types of carrageenan (κ, ι, and λ‐carrageenan) all demonstrate inhibitory effects on HPV, with half‐inhibitory concentration (IC_50_) values in the low ng/mL range. Notably, ι and λ‐carrageenan show more potent inhibitory effects compared to κ‐carrageenan (ι‐carrageenan: IC_50_: 0.006 μg/mL, 95% CI: 0.0050–0.0063 μg/mL; λ‐carrageenan: IC_50_: 0.010 μg/mL, 95% CI: 0.0093–0.011 μg/mL; κ‐carrageenan: IC_50_: 0.044 μg/mL, 95% CI: 0.037–0.052 μg/mL) [35]. Beyond blocking the interaction between HPV and heparan sulfate proteoglycan (HSPG), current findings suggest that carrageenan can also inhibit HPV infection through a secondary mechanism that does not involve HSPG binding [35, 36]. In 2014, Wang et al. demonstrated that carrageenan could inhibit furin‐cleaved pseudovirus (fcPsV) infection in HSPG‐deficient cell lines, indicating that carrageenan might inhibit HPV infection by blocking the surface of viral particles, preventing their binding to secondary receptors, rather than preventing the initial conformational changes leading to L2 exposure and furin cleavage (Figure 2) [36]. However, the HSPG‐independent inhibition still needs further investigation.

Currently, many in vitro studies have confirmed carrageenan's significant inhibitory effect on HPV [35, 37, 38, 39, 40, 41]. In 2007 and 2011, Roberts et al. further validated carrageenan's ability to inhibit HPV infection in mouse and primate models [42, 43]. Subsequent clinical studies have reinforced its potential as a promising therapeutic agent against HPV infection [41, 44, 45, 46, 47]. In 2023, a randomized, placebo‐controlled IIB trial by Cassandra Laurie et al. evaluated the efficacy and safety of carrageenan gel in preventing HPV infection. From January 16, 2013, to September 30, 2020, a total of 461 healthy women aged ≥ 18 years were randomly assigned to the carrageenan gel group (*n* = 227) or the placebo gel group (*n* = 234). Participants were instructed to apply gel on their own every other day for the first month and before/after vaginal intercourse (approximately 5–10 mL of gel applied to the vagina, penis and/or condom). The researchers collected vaginal samples and questionnaire data from the participants at each visit (0, 0.5, 1, 3, 6, 9, 12 months). Compared with 66.5% (147/221) of subjects in the placebo group who obtained ≥ 1 human papillomavirus type, the probability of the carrageenan group was only 51.9% (108/208), with a hazard ratio of 0.63 (95% CI: 0.49–0.81), *p* = 0.0003, indicating a significant reduction in the risk of viral infection, without an increase in adverse reactions [47]. In summary, carrageenan is undoubtedly a promising anti‐HPV drug.

### 1.8 Defensins

Defensins are cationic antimicrobial and antiviral peptides which are prevalent in both female and male reproductive tracts [48]. They play a critical role in the innate immune system [49]. Defensins are categorized into α‐defensins, β‐defensins, and θ‐defensins based on their structure and functional characteristics [48]. Humans express two types of defensins, α‐ and β‐defensins, while θ‐defensins are found in the white blood cells of rhesus monkeys [48, 49, 50, 51]. They exhibit antiviral properties against a range of viruses, including both enveloped and nonenveloped types [49, 51, 52]. In 2006, Christopher B. Buck et al. found that, apart from human defensin‐6 (HD‐6), which does not inhibit human papillomavirus 16 pseudoviruses (HPV16 PsV) at non‐cytotoxic levels, five out of the six human α‐defensins, such as human neutrophil peptide1‐4 (HNP1‐4) and human defensin‐5 (HD‐5), are effective inhibitors of PsV transduction. Notably, HD‐5 is particularly effective in inhibiting HPV, with an IC_50_ in the high ng/mL range (IC_50_: 0.6 μg/mL, 95% CI: 0.55–0.75 μg/mL). In stark contrast, the anti‐HPV activity of human β‐defensins 1 and 2 is minimal or absent (HBD‐2, IC_50_: 190 μg/mL, 95% CI: 178–203 μg/mL) [10]. In 2012, a study indicated that the anti‐HPV effect of HD‐5 is associated with its arginine residues [53]. Subsequently, a 2014 study further validated the inhibitory effect of HD‐5 on HPV infection. The data from this study indicate that human HD‐5 can dose‐dependently inhibit HPV16 pseudovirus infection in cells such as Ect 1, C33‐A, and CaSki, and can exert its effect at concentrations as low as 0.01 μg/mL [54].

**FIGURE 3:**
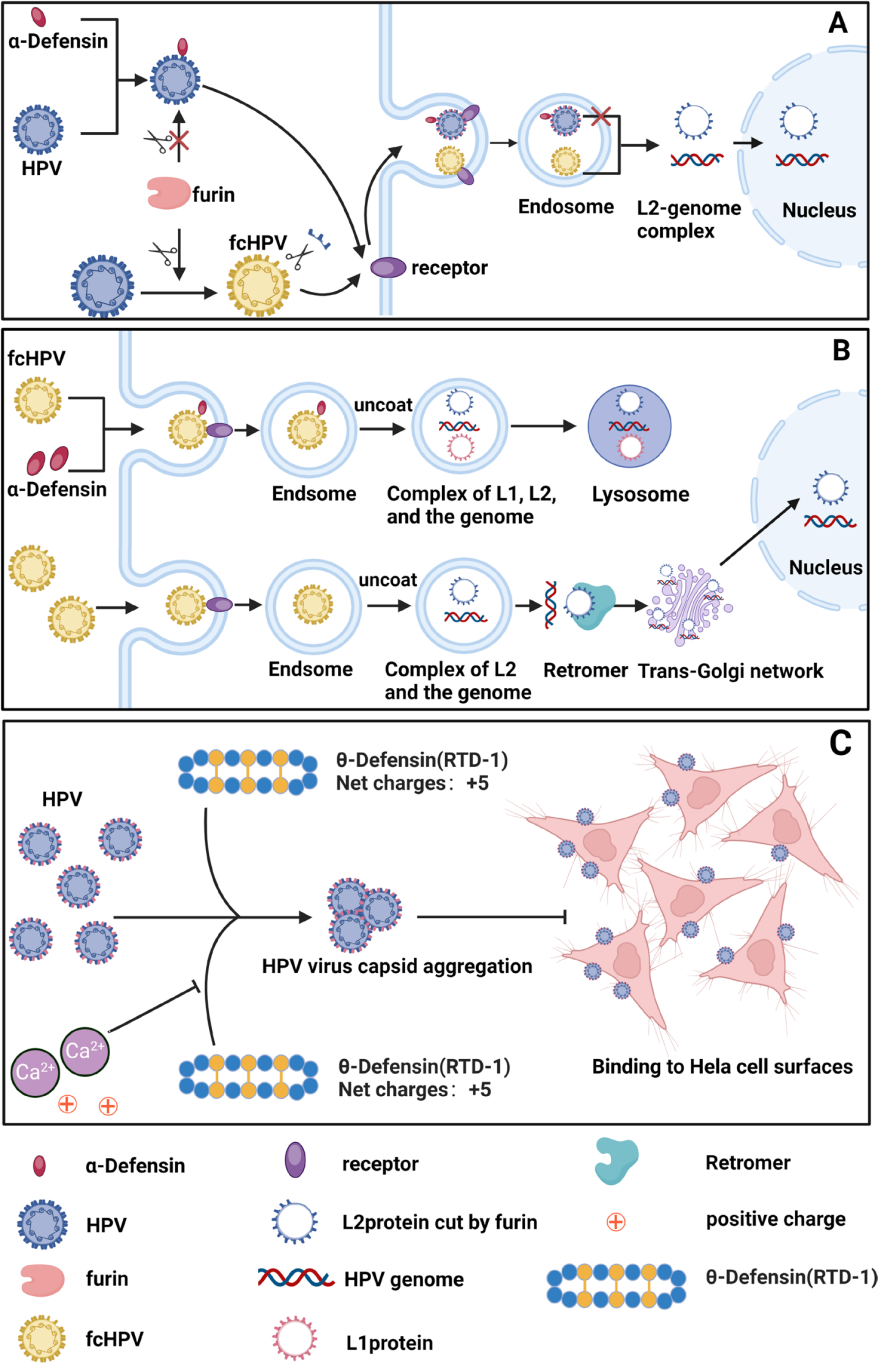
The mechanisms of several defensins inhibiting HPV infection. (A) First possible schematic diagram of HD‐5 inhibiting HPV infection. HD‐5 binds to the L1 protein of HPV, creating a spatial hindrance that limits the ability of furin protease to enter L2. furin protease is unable to cleave the L2 protein to form fcHPV. Due to the lack of furin protease cleavage, L1 and L2 cannot dissociate, and the virus cannot enter the nucleus in the form of L2 genome complex, but is left in the endosome. (B) Second possible schematic diagram of HD‐5 inhibiting HPV infection. For HPV viruses with the L2 protein cleaved by furin protease (fcHPV), HD‐5 can still stabilize the capsid by binding to HPV, preventing the virus from completely uncoating and preventing the separation of L1 and L2. This allows the virus to exist in the endosome in the form of L1, L2, and genome complexes, and ultimately be transported to lysosomes instead of L2 genome complexes, where it interacts with reverse transcriptase and is transported to the nucleus by the trans Golgi network. (C) Schematic diagram of θ‐defensins inhibiting HPV infection. The θ‐defensins significantly reduce HPV uptake by aggregating the viral capsid through their intrinsic charge (+5 charge), thereby inhibiting the initial binding of viral particles to the cell surface. If an excessive amount of divalent cations (such as Ca^2+^) are added, it will prevent the viral aggregation of θ‐defensins.

In 2015, Wiens and colleagues introduced a novel mechanism through which HD‐5 inhibits HPV infection [52]. The entry of HPV into cells occurs via endocytosis, necessitating the escape from the endosome into the cytoplasm, a process that requires the cleavage of the capsid protein L2 at a specific site recognized by furin protease [55]. Wiens et al. demonstrated that HD‐5 can block infection by inhibiting the cleavage of furin protease. The specific mechanism involves HD‐5 binding to HPV, creating steric hindrance that prevents furin protease from accessing the cleavage site on L2, rather than directly diminishing the activity of furin protease. At the same time, researchers hypothesize that the interaction may involve a specific binding site between HD‐5 and the viral L1 protein (Figure 3A) [52]. Key steps in HPV infection include: (1) the dissociation of L1 from L2 and the viral genome, (2) the transport of L2 and the viral genome to the trans Golgi network [56, 57]. In 2017, the research team proposed another possible mechanism. This study suggests that HD‐5 stabilizes the viral capsid by directly binding to it, thereby preserving its relative integrity and preventing the dissociation of L1 from L2 and the viral genome. At the same time, by preventing the interaction between L2 and reverse transcriptase in the cytoplasm, reverse transcriptase‐mediated retrograde transport of vesicles containing L2 and viral genome from the endosome to the trans Golgi network is blocked. The viral genome is directed towards lysosomes and accelerates the degradation of L1 and L2 (Figure 3B) [58].

In addition to α‐defensins, other studies have found that θ‐defensins can also inhibit HPV infection. The mechanism involves aggregating the HPV capsid, thus obstructing the binding of viral particles to the cell surface. This aggregation is related to the positive charge of θ‐defensins themselves (Figure 3C) [7]. However, the challenges associated with synthesizing θ‐defensins significantly limit their clinical application. Fortunately, by eliminating the cyclic backbone, the innovative peptide that simulates the humanized θ‐defensins structure can be produced in large quantities using standard chemical methods. Additionally, it exhibits antiviral properties that are on par with those of θ‐defensins, potentially paving the way for its future clinical applications [59].

### 1.9 25‐Hydroxycholesterol

**FIGURE 4:**
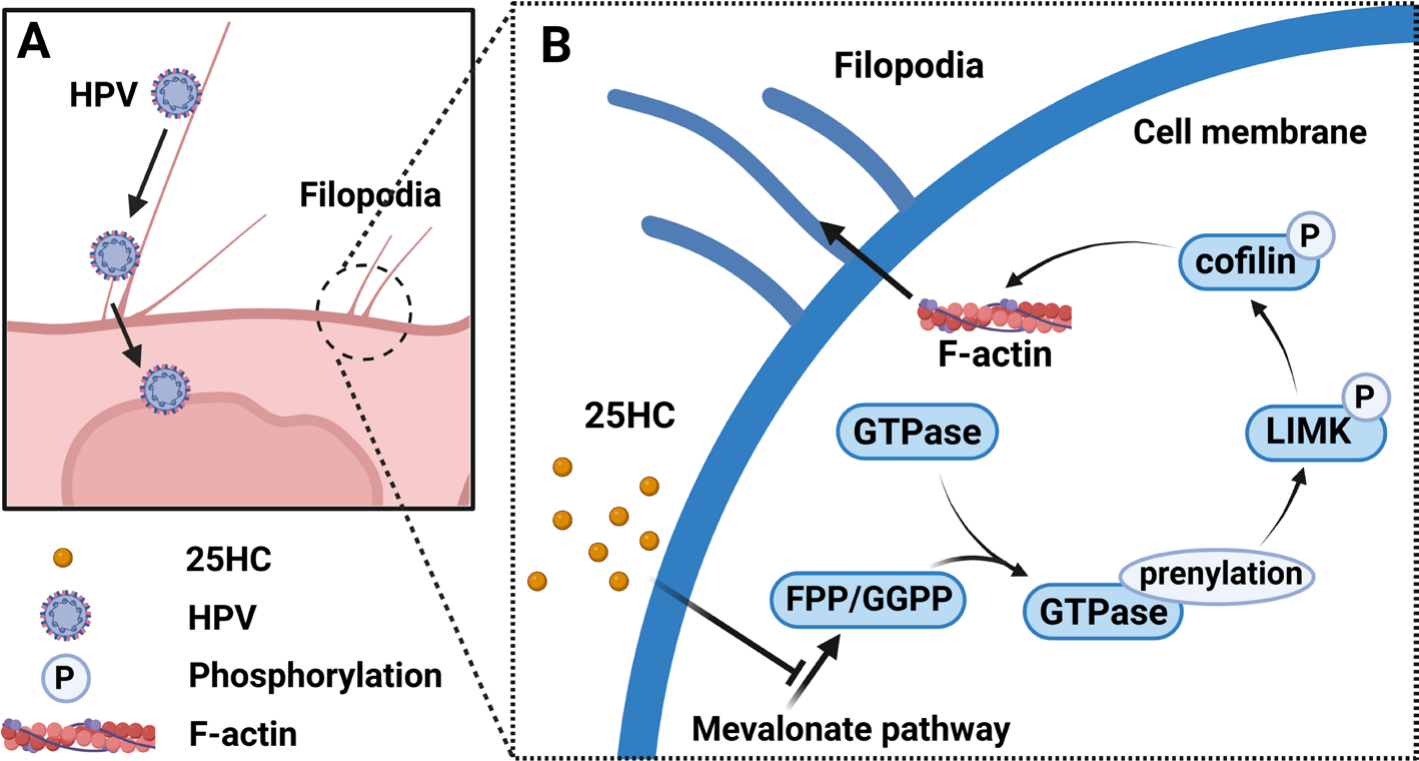
The mechanism of 25‐Hydroxycholesterol inhibiting HPV infection. (A) Actin inversely transports the virus to cells and infects cells. (B) 25HC inhibits the synthesis of isoprenoid substrates FPP and GGPP by suppressing the mevalonate (MVA) pathway, thereby preventing small GTPases from isoprenoidizing and inhibiting phosphorylation of LIMK/cofilin, interfering with Actin remodeling, and preventing the formation of filamentous pseudopodia. In this way, HPV cannot enter cells in a “viral particle surfing” manner along the filamentous pseudopodia. Ultimately, the HPV infection is suppressed.

During the lifecycle of viruses, cholesterol metabolism in cells is crucial. The adsorption, entry, assembly, budding, and release of some viruses often occur in cholesterol rich areas of the cell membrane [60]. Viruses possess specific lipid microenvironment requirements that are essential for various stages of their lifecycle [61]. Inhibiting the biosynthesis of cholesterol and fatty acids can elicit antiviral responses against various viruses [62, 63, 64, 65, 66]. As a steroid pathway inhibitor, 25‐Hydroxycholesterol (25HC) can demonstrate antiviral properties by inhibiting cholesterol synthesis. It has been shown to have inhibitory effects on various enveloped and nonenveloped viruses [9, 67, 68, 69, 70]. Its antiviral mechanisms may involve altering cell membrane characteristics, interacting with hydroxysterol binding proteins, inhibiting protein isopentenylation, integrating stress responses, enhancing interferon signaling, affecting inflammation, and adaptive immunity [71]. In 2014, a study exploring the effect of 25HC on nonenveloped viruses first highlighted that 25HC has significant antiviral activity against HPV16 [half maximal effective concentration (EC_50_) of 2.20 μM (95% CI: 1.61–2.99)] [9]. In 2023, Li et al. further investigated the anti HPV effect of 25HC and found that it acts as a broad‐spectrum anti‐HPV agent, effectively inhibiting high‐risk HPV types (such as HPV16, HPV18, and HPV59), HPV that may cause cancer (such as HPV73), and low‐risk HPV (such as HPV6) [72]. At the same time, they found that the specific mechanism by which 25HC inhibits HPV may be by reducing the production of isoprenoids in the mevalonate (MVA) pathway, limiting the isopentenization of small GTPase, and consequently inhibiting their post‐translational modification. Since small GTPase is upstream of LIMK/cofilin, the phosphorylation of LIMK/cofilin is inhibited, interfering with actin remodeling and preventing the formation of filamentous pseudopodia. In this way, HPV cannot enter cells in a “viral particle surfing” manner along the filamentous pseudopodia. Ultimately, the HPV infection is suppressed (Figure 4) [72, 73]. Although further research is needed to explore the deeper mechanisms and safety of 25HC against HPV, its established antiviral effects make it a promising candidate for cervical cancer prevention.

#### 1.9.1 Others

In addition to the above drugs, some potential drugs that can be used to combat HPV infection have been reported one after another. Curcumin, for instance, is a potent HPV inhibitor that reduces the growth and motility of HPV‐positive cervical cancer cells in a dose‐dependent manner, significantly decreasing colony formation (*p* < 0.05) and cell motility (*p* < 0.005) at concentrations of 5 and 10 μM. It can also induce apoptosis in cervical cancer cells via caspase‐mediated signaling [74, 75]. Juglone has been shown to inhibit the growth of HPV‐positive cervical cancer cells in a dose‐dependent and time‐dependent fashion, effectively reducing cell viability at 10 μM (*p* < 0.05), while not affecting HPV‐negative cervical cancer cells at the same concentration [76]. A study investigating the effect of androgens and their derivatives on HPV16 pseudovirus infection in cervical cancer cells found that androgens and their derivatives can inhibit HPV infection by binding to host cell receptors to prevent the binding of HPV16 pseudovirus to host cell receptors. Among these, 14‐deoxy‐11,12‐didehydrated andrographolide (14‐DDA) exhibits the highest efficacy [77]. Another investigation into the in vitro inhibitory effects of monoterpenoid zinc tetra‐ascorbo‐camphorate on HPV reported strong inhibitory activity against HPV16, particularly affecting the adsorption of HPV16 pseudovirus on COS‐7 cells, with an IC_50_ ranging from 2.9 to 8.3 μM [78]. Besides, pine needle‐methylene chloride (PN‐MC), polyethyleneimine, heparin, protamine sulfate (PS), guanylate binding protein 1 (GBP1), N,N′‐bisheteryl derivative of dispirotripiperazine (DSTP27), agmatine‐containing poly polymer (AGMA1) and bovine lactoferrin (BLF) have been proven by different scholars to have anti‐HPV infection activity [79, 80, 81, 82, 83, 84, 85, 86].

## 2 Conclusions

At present, antiviral drugs for the prevention and treatment of HPV are relatively scarce worldwide. In response, researchers from different countries have undertaken extensive research and found that various substances, including 3HP‐β‐LG, carrageenan, defensins, and 25HC, have strong inhibitory effects on HPV (Table 1). These findings suggest the potential development of new types of inhibitors for HPV infection.

**TABLE 1:** Inhibitory effects of various drugs.

Drug	Virus	Cell	IC_50_	95% CI	Cytotoxicity	Source
3‐hydroxyphthalic anhydride‐modified bovine β‐lactoglobulin (3 HP‐β‐LG)	HPV6 PsV	293FT	0.33 μM		> 1 mg/mL	[23]
	HPV16 PsV	293FT	0.04 μM		> 1 mg/mL	
	HPV18 PsV	293FT	0.065 μM		> 1 mg/mL	
	HPV16 PsV	HeLa	0.59 μg/mL			[26]
	HPV58 PsV	HeLa	0.28 μg/mL			
	HPV16 PsV	HaCaT	5.40 μg/mL			
	HPV58 PsV	HaCaT	0.47 μg/mL			
ι‐Carrageenan	HPV16 PsV	HeLa	0.006 μg/mL	0.0050 ~ 0.0063 μg/mL		[35]
ι‐Carrageenan, typeII	HPV16 PsV	HeLa	0.005 μg/mL	0.0042 ~ 0.0049 μg/mL		
ι‐Carrageenan, typeV	HPV16 PsV	HeLa	0.006 μg/mL	0.0044 ~ 0.0080 μg/mL		
ι‐Carrageenan, typeVa	HPV16 PsV	HeLa	0.004 μg/mL	0.0036 ~ 0.0046 μg/mL		
κ‐Carrageenan	HPV16 PsV	HeLa	0.044 μg/mL	0.037 ~ 0.052 μg/mL		
κ‐Carrageenan, typeIII	HPV16 PsV	HeLa	0.013 μg/mL	0.011 ~ 0.016 μg/mL		
κ/λ‐Carrageenan, typeI	HPV16 PsV	HeLa	0.021 μg/mL	0.020 ~ 0.022 μg/mL		
λ‐Carrageenan	HPV16 PsV	HeLa	0.010 μg/mL	0.0093 ~ 0.011 μg/mL		
λ‐Carrageenan, rypeIV	HPV16 PsV	HeLa	0.005 μg/mL	0.0039 ~ 0.0058 μg/mL		
human neutrophil peptide1 (HNP‐1)	HPV16 PsV	HeLa	5 μg/mL	3.20 ~ 6.60 μg/mL	> 0.10 mg/mL	[10]
HNP‐1 (Pepnet)	HPV16 PsV	HaCaT	9 μg/mL	3.70 ~ 18.60 μg/mL	> 0.10 mg/mL	
HNP‐1 (Pepnet)	HPV16 PsV	293TT	8 μg/mL	6.00 ~ 10.10 μg/mL	> 0.10 mg/mL	
HNP‐2 (American Peptide)	HPV16 PsV	HeLa	4 μg/mL	2.50 ~ 7.60 μg/mL	> 0.10 mg/mL	
HNP‐3 (Pepnet)	HPV16 PsV	HeLa	10 μg/mL	6.90 ~ 13.10 μg/mL	> 0.10 mg/mL	
HNP‐4	HPV16 PsV	HeLa	21 μg/mL	6.90 ~ 60.60 μg/mL	0.05 mg/mL	
human defentin‐5 (HD‐5)	HPV16 PsV	HeLa	0.60 μg/mL	0.55 ~ 0.75 μg/mL	> 0.10 mg/mL	
HD‐6	HPV16 PsV	HeLa	—	—	0.05 mg/mL	
Human β defensin‐1 (HBD‐1)	HPV16 PsV	HeLa	—	—	> 0.39 mg/mL	
HBD‐2 (Pepnet)	HPV16 PsV	HeLa	190 μg/mL	178 ~ 203 μg/mL	> 0.43 mg/mL	
HD‐5	HPV16 PsV	HeLa	1.10 μM	0.93 ~ 1.32 μM		[52]
Rhesus Theta Defensin‐1 (RTD‐1)	HPV16 PsV	HeLa	14 μM			[7]
RTD‐2	HPV16 PsV	HeLa	16.30 μM			
RTD‐4	HPV16 PsV	HeLa	101.90 μM			
25‐Hydroxycholesterol (25HC)	HPV6 PsV	HeLa	(2.86 ± 0.44) μM			[72]
	HPV16 PsV	HeLa	(0.92 ± 0.12) μM			
	HPV18 PsV	HeLa	(0.80 ± 0.10) μM			
	HPV59 PsV	HeLa	(0.36 ± 0.06) μM			
	HPV73 PsV	HeLa	(3.55 ± 0.40) μM			
	HPV6 PsV	C‐33A	(1.33 ± 0.40) μM			
	HPV16 PsV	C‐33A	(2.34 ± 0.34) μM			
	HPV18 PsV	C‐33A	(5.66 ± 0.31) μM			
	HPV59 PsV	C‐33A	(1.67 ± 0.35) μM			
	HPV73 PsV	C‐33A	(2.58 ± 0.68) μM			
Heparin	HPV16 PsV	HeLa	1.3 μg/mL	1.3 ~ 1.4 μg/mL		[35]
Zinc tetra‐ascorbo‐camphorate	HPV‐16 PsV	COS‐7	2.90 ~ 8.30 μM			[78]
Protamine Sulfate (PS)	HPV‐5 PsV	HaCaT	0.078uM			[82]
	HPV‐6 PsV	HaCaT	0.33 μM			
	HPV‐16 PsV	HaCaT	0.096 μM			
	HPV‐18 PsV	HaCaT	0.21 μM			
	HPV‐31 PsV	HaCaT	0.073 μM			
	HPV11 QV	HaCaT	0.64 μM			
	HPV16 QV	HaCaT	10.2 μM			
	HPV31 QV	HaCaT	1.71 μM			
N,N′‐bisheteryl derivative of dispirotripiperazine (DSTP27)	HPV16 PsV	293TT	1.4 μM			[84]
	HPV18 PsV	293TT	0.7 μM			
Agmatine‐containing poly polymer (AGMA1)	HPV16 PsV	293TT, SEAP	0.53 μg/mL	0.51 ~ 0.54 μg/mL		[85]
	HPV16 PsV	293TT, GFP	0.38 μg/mL	0.30 ~ 0.48 μg/mL		
	HPV16 PsV	HeLa	0.38 μg/mL	0.28 ~ 0.52 μg/mL		
	HPV16 PsV	SiHa	0.38 μg/mL	0.34 ~ 0.42 μg/mL		
	HPV16 PsV	C33A	0.49 μg/mL	0.38 ~ 0.63 μg/mL		
	HPV31 PsV	293TT	0.36 μg/mL	0.28 ~ 0.46 μg/mL		
	HPV45 PsV	293TT	0.74 μg/mL	0.70 ~ 1.80 μg/mL		
	HPV6 PsV	293TT	0.54 μg/mL	0.36 ~ 0.81 μg/mL		
Bovine lactoferrin (BLF)	HPV16 VLP	HaCaT	35 μg/mL			[86]

*Note:* (−) No inhibitory effect was observed at the highest cytotoxic dose.

Abbreviations: 25HC, 25‐hydroxycholesterol; 3 HP‐β‐LG, 3‐hydroxyphthalic anhydride‐modified bovine β‐lactoglobulin; AGMA1, agmatine‐containing poly polymer; BLF, bovine lactoferrin; CI, confidence interval; DSTP27, N,N′‐bisheteryl derivative of dispirotripiperazine; HBD, human β defensin; HD, human defentin; HNP, human neutrophil peptide; IC_50_, half maximal inhibitory concentration; PS, protamine sulfate; RTD, rhesus theta defensin.

The biological excipient composed of 3HP‐β‐LG has been clinically validated for safety and efficacy, exhibiting IC_50_ values in low micromolar ranges and showing no cytotoxicity at 1 mg/mL (Table 1). Carrageenan is a potent anti‐HPV agent, with efficacy three orders of magnitude greater than heparin, which is also considered a potent HPV inhibitor (Table 1). Multiple defense factors have also shown significant anti‐HPV activity, among which HD‐5 has the strongest antiviral activity, with an order of magnitude higher activity compared to HNPs 1–3 (Table 1). Furthermore, 25HC has broad‐spectrum anti‐HPV activity and has significant inhibitory effects on various HPV types including HPV6, HPV16, HPV18, HPV59, and HPV73. Its IC_50_ values are also at low micromolar concentrations (Table 1). With further research on it, it is expected to be used as a broad‐spectrum anti‐HPV agent for clinical prevention and treatment of cervical cancer. However, due to some studies lacking molecular weight information, many drugs listed in the table are still measured in μg/mL. Although the IC_50_ of carrageenan is not quantified in μM, its molecular weight of approximately 788 indicates that it has the lowest IC_50_ among the drugs listed (0.0076 μM). This is followed by 3HP‐β‐LG (0.04 μM), 25HC [(0.92 ± 0.12) μM], and HD‐5 (1.10 μM). These values can serve as a useful reference for assessing the relative strength of its anti‐HPV effects.

The clinical use of biological excipients containing 3HP‐β‐LG offers a novel avenue for cervical cancer prevention but comes with challenges such as high costs and extended treatment durations. Addressing how to lower expenses and streamline treatment timelines is an urgent issue. While carrageenan, defensins, and 25HC have shown inhibitory effects on HPV, their application in clinical prevention still requires further investigation to establish their effectiveness and safety. Additionally, there is a need for ongoing discussions on how to allocate these treatments effectively to fill the void left by vaccination efforts.

Safeguarding and promoting women's reproductive health is a crucial responsibility for governments and healthcare workers around the world. Cervical cancer prevention and treatment for women in developing countries remains particularly important. The strategy targeting HPV presents a fresh perspective for the prevention and treatment of cervical cancer and may become an essential and standard prevention and treatment measure in the future. In conclusion, this article presents a comprehensive overview of the latest advancements in the research of promising anti‐HPV medications, aiming to inspire novel strategies for the prevention and treatment of cervical cancer.

## Author Contributions

**Hangdi Chen:** conceptualization (lead), data curation (equal), investigation (equal), project administration (equal), software (equal), validation (equal), visualization (equal), writing – original draft (lead). **Kai Guo:** conceptualization (equal), investigation (equal), validation (equal), writing – original draft (equal). **Zhihao Bai:** conceptualization (equal), data curation (equal), investigation (equal), software (equal), validation (equal), visualization (equal). **Liuyi Lu:** investigation (lead), validation (equal). **Bin Liu:** software (equal), validation (equal). **Jiali Zhang:** data curation (equal), validation (equal). **Meiyin Zhong:** data curation (equal), validation (equal). **Changfen Xu:** validation (equal), visualization (equal). **Wanghuan Chen:** validation (equal), visualization (equal). **Aiwu Huang:** funding acquisition (equal), supervision (equal), validation (equal), writing – review and editing (equal). **Yuemin Ding:** conceptualization (lead), funding acquisition (equal), supervision (equal), validation (equal), writing – review and editing (lead).

## Conflicts of Interest

The authors declare no conflicts of interest.

## Data Availability

The authors have nothing to report.
